# Comparative Analysis of Fall Risk Assessment Features in Community-Elderly and Stroke Survivors: Insights from Sensor-Based Data

**DOI:** 10.3390/healthcare11131938

**Published:** 2023-07-05

**Authors:** Chia-Hsuan Lee, Tomas Mendoza, Chien-Hua Huang, Tien-Lung Sun

**Affiliations:** 1 Department of Data Science, Soochow University, No. 70, Linxi Road, Shilin District, Taipei 111, Taiwan; sweat0430@mail.ntust.edu.tw; 2 Department of Industrial Engineering and Management, Yuan Ze University, 135 Yuan Tung Road, Chungli District, Taoyuan 320, Taiwan; s1078907@mail.yzu.edu.tw; 3 Department of Eldercare, Central Taiwan University of Science and Technology, Taichung 40601, Taiwan; 108184@ctust.edu.tw

**Keywords:** fall risk, community-dwelling, stroke-survivors, random forest, feature selection, inertial sensor

## Abstract

Fall-risk assessment studies generally focus on identifying characteristics that affect postural balance in a specific group of subjects. However, falls affect a multitude of individuals. Among the groups with the most recurrent fallers are the community-dwelling elderly and stroke survivors. Thus, this study focuses on identifying a set of features that can explain fall risk for these two groups of subjects. Sixty-five community dwelling elderly (forty-nine female, sixteen male) and thirty-five stroke-survivors (twenty-two male, thirteen male) participated in our study. With the use of an inertial sensor, some features are extracted from the acceleration data of a Timed Up and Go (TUG) test performed by both groups of individuals. A short-form berg balance scale (SFBBS) score and the TUG test score were used for labeling the data. With the use of a 100-fold cross-validation approach, Relief-F and Extra Trees Classifier algorithms were used to extract sets of the top 5, 10, 15, 20, 25, and 30 features. Random Forest classifiers were trained for each set of features. The best models were selected, and the repeated features for each group of subjects were analyzed and discussed. The results show that only the stand duration was an important feature for the prediction of fall risk across all clinical tests and both groups of individuals.

## 1. Introduction

Falls are problems that affect different groups of individuals, and most studies on fall risk focus on older adults [1]. However, stroke survivors also experience falls. Given that these two categories of people fall frequently, it is crucial to create effective fall prevention programs since the expenses associated with falls place an increasing burden on the public health system [2].

Previous studies have determined that falls are a multifactorial problem [3]. Mobility [4], gait instability [5,6,7], and balance issues [4,7,8] are some of the most common causes that affect older adults. Similarly, the most common factors that affect stroke patients include balance [9,10,11,12] and mobility issues [11,13]. In response, fall-risk prevention programs use clinical tests to detect subjects who suffer from these issues. Two of the most common clinical tests associated with fall-risk prevention are the Timed-Up and Go (TUG) test and the Berg balance scale (BBS), as they were developed and evaluated in several fall-risk assessment studies [14]. Despite their effectiveness, their implementation requires the presence and expertise of a medical professional. This has presented the opportunity for researchers to study the applications of wearable inertial devices as auxiliary tools to assist medical professionals with their light weight, portability, low cost [14], and ability to collect sensitive and reliable TUG data [15,16] which enables researchers to identify reliable parameters [17].

Researchers have identified features that can be used with statistical or machine-learning models to identify older adults at risk of falling [18], as well as post-stroke individuals at risk of falling [19] using data collected by inertial sensors from the TUG test. However, the features identified by these studies have no medical interpretation for medical professionals, which makes analyzing the underlying health problems related to these falls challenging. To address these limitations, a similar fall-risk assessment study used a wait-mounted accelerometer to estimate the BBS score of community dwelling elderly [20]. The features used had medical meaning and are easily interpreted by medical professionals. However, most features extracted in this study require extensive signal processing and data cleaning, which makes the procedure difficult to reproduce in elderly homes without the constant monitoring of trained personnel. Moreover, none of the studies implemented a multifactor clinical test, which is more efficient at capturing the complex nature of falls [21,22].

Finally, to our knowledge, no research has analyzed the similarities between community-dwelling elderly adults and post-stroke patients that can predict fall risk. Our motivation to include both groups of subjects is due to their frequency of fall, the severity of the injuries they suffer because of their condition, and the limited number of studies analyzing features related to falls among post-stroke subjects. We will calculate numerous features from the TUG signals of all individuals. Using feature selection algorithms, we will estimate feature importance and compare them between both groups of subjects. Consequently, this study will focus on summarizing the features required to detect the largest number of classes with a higher potential to fall while simultaneously using machine-learning algorithms to discuss the benefits of automatic screening.

## 2. Materials and Methods

### 2.1. General Approach

Subjects from both groups wore an inertial sensor while they performed the TUG test. The data will be segmented into sit to stand (Sist), walk, turn, and stand to sit (Stsi), after which a set of features were calculated from the acceleration data of each subject. The scores for the TUG and SFBBS clinical tests were used as labels for the features. Using ETC and Relief-F algorithms, feature importance was calculated from both groups. A random forest classifier (RF) was used to classify the subjects into fall risk or healthy using different sets of features selected based on their importance. Finally, the most important features were analyzed to determine whether any features could be regarded as important for fall-risk prediction, independent of the type of subject being tested.

### 2.2. Subjects

The community elderly and stroke survivors both commonly use an inertial sensor to identify fall risk. This study focuses on identifying a set of features that can explain fall risk for subjects of these two groups.

#### 2.2.1. Community-Dwelling Elderly

Community-dwelling elderly subjects from a hospital in central Taiwan participated in a set of clinical tests between April 2014 and May 2015. The studies involving human participants were reviewed and approved by Tsaotun Psychiatric Center, Ministry of Health and Welfare (IRB No. 104013). A team of physiotherapists and rehabilitation physicians assisted and monitored the participants. All subjects wore a waist-mounted inertial sensor while completing the clinical assessments. As summarized in Table 1, data were collected from 65 elderly adults (with an average age of 76 ± 7 years). Such subjects were recruited after confirming that they had no history of musculoskeletal injuries or central nervous system injuries and that they could walk without aid to perform the clinical tests.

#### 2.2.2. Stroke Survivors

Between April 2018 and October 2018, we recruited stroke survivors from a hospital in north Taiwan to participate in a series of clinical tests (IRB No. TYGH106045). Subjects capable of performing these tests with or without walking assistance were included in this study. In total, we gathered data from 35 different individuals (22 men and 13 women) who had suffered from an Ischemic stroke. All subjects that participated in our study did it willingly and provided consent to have their acceleration data collected. A summary of the demographic data for the stroke survivors in our study is shown in Table 1.

### 2.3. Clinical Tests

In this research, two different clinical tests were performed by all test subjects, i.e., the short-form berg balance scale (SFBBS) and the TUG. The SFBBS was conducted by a professional physiotherapist. Subjects who took the SFBBS test were required to perform seven different activities, which are standing still with both eyes closed, sitting to standing transitions, standing with both feet while keeping an arm reaching forward, picking up an object from the floor, turning 360 degrees while standing up, standing with one foot in front, and standing on one leg unsupported. The professional physiotherapist assigned a score to each task performed by the subjects. This score ranged from 0 (subject could not perform the task) to 4 (subject performed the task without problems). Consequently, subjects who had no problems performing any of the seven tasks obtained the maximum score of 28. In contrast, subjects who had problems regarding their static balance obtained a score lower than 23, which was found to be the significant threshold to patients with posture problems by a previous study [23].

When performing the TUG test [24], subjects began by sitting on a chair. Then, they were asked to stand up, walk at a natural pace forward, turn 180 degrees when they reached a mark on the floor, walk back toward the chair, and sit down.

In this study, we used an inertial sensor to collect data from subjects when they performed the TUG test. We did not collect any acceleration data from the SFBBS test. Therefore, the features used in this study were extracted exclusively from the TUG acceleration signals of subjects. Moreover, we used the scores of the SFBBS test and the score of the TUG test to label the subjects as fallers and non-fallers. The duration of the TUG test was used to classify those subjects who performed the test in over 12.47 s as fallers, since a previous study found that this threshold was substantial for community-dwelling elderly [23]. Similarly, we labeled subjects with a SFBBS score lower than 23 as fallers, as this was also substantial in previous studies [20,25].

### 2.4. Wearable Accelerometer

**Figure 1:**
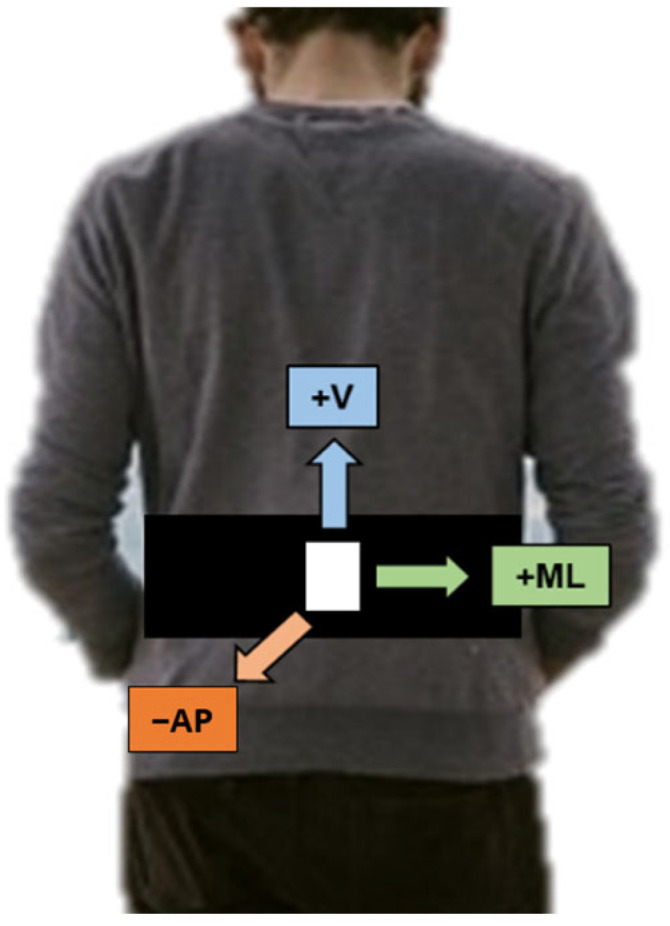
Illustration showing the estimated location of the inertial sensor (white box). This sensor was attached to a belt, and it was located on the lower back of subjects (between the L4 and L5 vertebrae). The three axes of the sensor collected along with their orientations are shown for reference.

To find a set of features that are not related to the type of sensor used, different sensors were used to collect data from each subject group. For the community-dwelling elderly, the ADXL345 accelerometer was used. This sensor collected data at a frequency of 30 Hz from three different axes, namely mediolateral (ML), vertical (V), and anterior–posterior (AP). For the stroke survivor subjects, a triaxial accelerometer (RD3152MMA7260Q, Freescale Semiconductor-NXP, Austin, TX, USA) sensor was used. It was calibrated at a frequency of 45 Hz and recorded acceleration data from the ML, V, and AP axes. Each sensor was attached to a waist-mounted strap, and it was situated at the lower back of the subjects. This location approximates the center of mass of most individuals, making it the most common across similar studies [14]. An illustration of the location of the sensors for both experiments can be found in Figure 1.

### 2.5. Data Analysis

#### 2.5.1. Feature Extraction

We calculated a set of 79 different features from the inertial sensor data using Python, which can be found in Table 2. Every feature was calculated for each axis (ML, V, and AP), and was found to be related to fall risk by previous studies. This section introduces these features from a physiological point of view.

Root mean square (RMS) represents the degree of spread of the data with respect to zero [26]. As the data in this study were collected from the lower back level, RMS measures the degree of variability in trunk acceleration. This feature is commonly used in similar studies, as maintaining balance relies heavily on trunk control since this the approximate location of center of body mass [27,28]. Consequently, previous studies have found low acceleration RMS to be associated with instability [29,30,31], which directly affects posture.

Similar to RMS, jerk measures the rate of change in acceleration [32]. Jerk is a common feature in previous studies as healthy subjects will exert higher muscle strength when performing sit to stand or stand to sit transitions [33], which may result in noticeable acceleration changes. These acceleration changes can also be reflected in subjects as they lean forward during standing or backwards while sitting and can be captured by their maximum acceleration values [34,35]. Moreover, subjects with posture balance problems perform the standing and sitting transitions in a more controlled manner as they have reduced ability to control their movement while performing these tasks. This restricted movement has been captured by previous studies and found to be considerably different between fallers and non-fallers as shown by their standard deviation measurements of acceleration [36,37], median acceleration values [37], and range acceleration values [37].

Individuals who are at risk of falling also exhibit abnormal sways when walking [27,38]. This abnormal sway can be caused by a strategy of remaining in control of their balance to avoid falling [39]. Consequently, this strategy of caution affects the total time it takes subjects to walk. In fact, studies have found walk duration to be a good predictor of falls walk duration [40,41,42,43]. A similar strategy used by frail subjects at risk of falling is to take smaller steps to improve their balance while walking, as reflected by shorter step lengths [44,45,46] and stride length [43,47]. Reducing walking speed is also common, as shown by recent studies that found significant relations between risk of falling and gait speed [43,47], cadence [43,47], stride time [48], and step time [48].

Stride length measures the distance from the moment a particular heal touches the ground, goes through a gait cycle, and touches the ground again. Similarly, step length measures the distance from the moment a heel touches the ground to the moment the heel on the opposite side touches ground, which is usually half of a stride. As observed by previous studies, stroke survivors suffer from variations in step and stride length caused by underlying paretic leg impairment [49,50]. These differences provide information on the severity of gait abnormalities and have implications for fall risk assessment. Understanding these differences and analyzing their relationship with fall risk can provide comprehensive information for stroke patient fall risk assessment and help to screen or design more effective interventions. Similar variations are also common in elderly subjects with dementia [51]. These abnormalities in gait increase the risk of falling, as observed by recent studies which found subjects at risk of falling to have higher coefficient of variation (CV) for step time and stride time [51,52,53] when compared to healthy subjects.

Postural problems can also be identified during the standing and sitting transitions. Sit-to-stand and stand-to-sit durations have been found to be statistically significant between healthy elderly and those with transitional posture problems [54,54,55,56]. Sit-to-stand duration has also been found to be statistically significant among stroke survivors as they need considerably more time to achieve stability when standing up [57]. Similarly, stand-to-sit duration was also found to be a good predictor of falls among stroke survivors, as subjects tend to shift their weight towards one leg, which causes difficulties to sit naturally [57].

#### 2.5.2. Multiscale Entropy (MSE) Analysis

**Figure 2:**
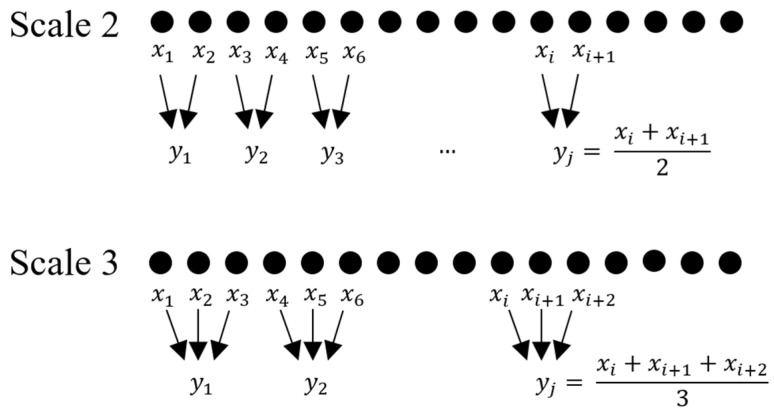
Illustration of the calculation steps for obtaining the coarse-grained series from an original signal at two different scales. Figure originally available in a previous study [58].

The calculation of MSE begins by defining the scaling factors τ to be analyzed. Then, for each scaling factor, a coarse-grained series is extracted from a given time series of length N. This process is performed by estimating the mean of all data points within a sliding window of size τ. As the name suggests, this window slides through the entire time series; thus, the resulting coarse-grained series has a length of N/τ data points. An example found in another study [58] illustrating the process of calculating coarse grained series can be found in Figure 2.

Next, for each coarse-grained series, sample entropy (SampEn) is calculated. SampEn measures the complexity of a signal by finding the probability that similar sequences of m consecutive data points will remain similar if their number of data points increases by one data point. As observed in Equation (1), a signal with low complexity has a SampEn value close to zero.

(1) $$SampEn=-ln\frac{C^{m+1}(r)}{C^{m}(r)}$$

Finally, after calculating SampEn, the complexity index (CI) is calculated as the sum of the SampEn values of all coarse-grained series (for all scaling factors τ), as illustrated in Equation (2). CI was found useful to categorize falling behavior [59], as it can measure the information contained in physiological time series over multiple scales.

(2) $$\sum_{\tau =1}^{n}SampEn(\tau )$$

#### 2.5.3. Permutation Entropy (PE)

The first step in calculating PE is to use a window (of length D) and slide it τ data points each step through the entire time-series data (of length T). This will result in a two-dimensional matrix of shape D × T − (D − 1) *τ*, where each column represents the data scanned at each step by the sliding window. To illustrate this process, consider the following example. Given a time series S(t)={8,5,4,3,11,9,1}, the dimensional matrix we would obtain if we used a sliding window D = 3 and *τ* = 1 is:

$$\left[\begin{array}{ccccc} 8 & 5 & 4 & 3 & 11\\ 5 & 4 & 3 & 11 & 9\\ 4 & 3 & 11 & 9 & 1 \end{array}\right]$$

Then, each column vector of the matrix is mapped into all possible permutations of itself. To achieve this, we first find all possible ordinal patterns which capture the ordinal rankings of the data. We can find these by calculating all possible permutations for a given window size. In this particular case, given our sliding window of size D = 3, then the ordinal patterns are

$$\pi_{1}=\{0,1,2\}\;\pi_{2}=\{0,2,1\}\;\pi_{3}=\{1,0,2\}\;\pi_{4}=\{1,2,0\}\;\pi_{5}=\{2,0,1\}\;\pi_{6}=\{2,1,0\}$$

To map these ordinal patterns to the matrix obtained above, it is necessary to observe the order of the values in each column. For example, given the first column, the permutation that should be mapped to it should be $\pi_{1}=\{2,1,0\}$, since 8 > 5 > 4. Therefore, if we map all the permutations to our matrix, we would obtain the following permutation matrix:

$$\left[\begin{array}{ccccc} 2 & 2 & 1 & 0 & 2\\ 0 & 0 & 0 & 2 & 1\\ 1 & 1 & 2 & 1 & 0 \end{array}\right]$$

Given the permutation matrix, the frequency of each permutation that appears throughout all sequences is then calculated. This frequency is then divided over the total number of sequences (or several columns in the matrix), which gives a probability p. For the permutation matrix obtained above, the probabilities $p_{i}$ of each ordinal pattern are

$$p_{\pi_{1}}=0/5\;p_{\pi_{2}}=1/5\;p_{\pi_{3}}=1/5\;p_{\pi_{4}}=0/5\;p_{\pi_{5}}=2/5\;p_{\pi_{6}}=1/5$$

Finally, the value for *PE* for a given order *D* is obtained using Equation (3):

(3) $${PE}_{D}=\sum_{i=1}^{D!}p_{i}{log}_{2}p_{i}$$

### 2.6. Feature Importance and Classification

**Figure 3:**
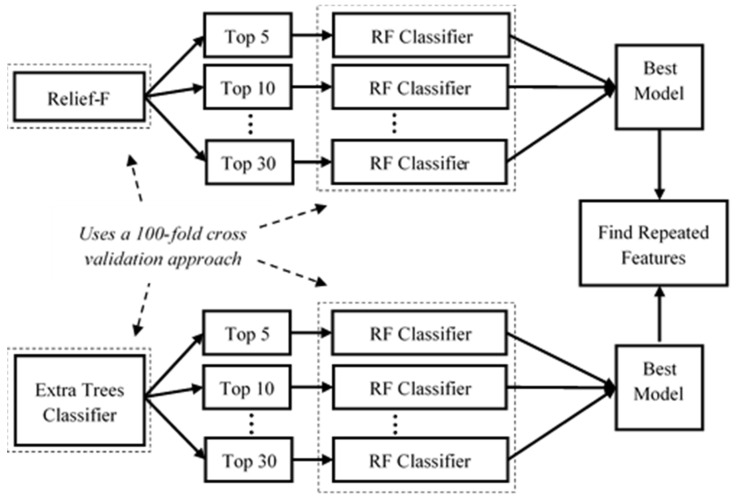
Illustration of the procedure to estimate important features.

The methodology proposed for feature importance selection is inspired by a recent and novel study [60]. Using the score and specific criteria for each clinical test, we labeled each subject as either fall risk or non-fall risk. With a 100-fold cross-validation strategy, we used two different feature selection algorithms, namely Relief-F and ETC, to find the top 5, 10, 15, 20, 25, and 30 features for each clinical test. We selected this number of folds as it is a common technique to reduce bias towards samples in small datasets [61]. Using each set of features, we used a random forest algorithm with a 100-fold cross-validation approach to classify subjects into fall-risk or healthy categories. Furthermore, we selected the best model for each clinical test, feature selection algorithm, and subject group, based on the average AUC score across folds. Finally, we selected those features found in both models as the set of important features for the respective clinical test. This entire procedure is illustrated in Figure 3.

#### 2.6.1. Relief-F

Relief-F has been used for feature selection in fall-risk assessment studies and gait analysis studies [60,62,63]. This popularity can be attributed to its numerous characteristics, such as its computational efficiency when dealing with large feature spaces (which are common in fall-risk assessment studies). It is capable of detecting feature dependencies by indirectly deriving interactions through the concept of nearest neighbors [64]. Furthermore, Relief-F is a non-parametric feature selection method, which allows it to determine feature importance across a wide range of datasets without relying on the underlying distribution of the data [65]. Contrary to other filter-based feature selection methods, Relief-F has more robustness against imbalanced datasets [65]. Thus, it has been preferred for our imbalanced dataset.

The main objective of this algorithm is to estimate the quality of attributes (features) based on their ability to classify samples that are similar. Features that can correctly classify neighboring samples obtain high-quality estimation, whereas features that misclassify neighboring samples are ranked poorly. This iterative algorithm starts at iteration i=1 by first setting the quality of all samples, $w_{j}$, to 0. Then, for each next iteration *i* = 1, 2, …, *m*, the algorithm randomly selects a sample, $x_{r}$, and using the Manhattan distance,$\;d_{rq,}$, it computes a set of k-nearest neighbors for each class. Finally, it updates the quality estimation, $w_{j}$, for each neighbor $x_{q}$ using Equation (4) assuming $x_{r}$ and $x_{q}$ belong to the same class or using Equation (5) if they belong to different classes.

(4) $$W_{j}^{i}=W_{j}^{i-1}-\frac{{∆}_{j}(x_{r},x_{q})}{m}\cdot d_{rq}$$

(5) $$W_{j}^{i}=W_{j}^{i-1}+\frac{p_{y_{q}}}{1-p_{y_{r}}}\cdot \frac{{∆}_{j}(x_{r},x_{q})}{m}\cdot d_{rq}$$

where $W_{j}^{i}$ is the weight of the feature $F_{j}$ at iteration $ip_{y_{r}}$, $p_{y_{q}}$ prior probability of $x_{r}$’s and $x_{q}$’s class, respectively, m is the maximum number of iterations, set by the user, and ${∆}_{j}$ is the difference of the feature value $F_{j}$ between $x_{r}$ and $x_{q}$, and is expressed as ${∆}_{j}=\frac{\left|x_{rj}-x_{qj}\right|}{{max(F}_{j})-{min(F}_{j})}$.

#### 2.6.2. Extra Trees Classifier (ETC)

Decision Trees (DT) are algorithms that classify samples by recursively evaluating features that best split the data. For categorical DT, this splitting criterion is determined using different metrics, i.e., the Gini index or entropy. The main problem with DTs is that they are inaccurate, and thus can be solved by random forests (RF) [66] by combining many DTs to make predictions. However, combining multiple DTs without any data preparation results in a highly biased prediction. Thus, RFs use bootstrapping to reduce correlation across trees. This technique consists of the generation of datasets of the same size as the original but with randomly selected samples with replacement. Then, for each bootstrapped dataset, a decision tree is created using only a random subset of features n at each step. Therefore, given a new sample, the classification results for all trees are aggregated (this technique is called bagging), and the final classification result is obtained. Finally, feature importance is calculated by estimating the average of each decrease in the impurity of the feature across trees.

ETC has also been used for feature selection in studies of fall-risk assessment [60,62,67]. Non-parametric in nature, ETCs serve as effective tools for uncovering nonlinear associations and are considered valuable in the analysis of data [68]. They achieve this by using randomized splitting points for each tree. Moreover, they are highly interpretable and can be used for both discrete and continuous data. Furthermore, they are able to reach a balanced ratio between variance and bias when compared to other feature selection algorithms [69]. ETCs do not use bootstrapped datasets but rather consider the whole dataset for each DT. In addition, they consider a random subset of features at each step when building a DT, and this subset is generally larger in ETC than in RF. Finally, the split decision at each node is random, as opposed to the impurity criteria used by RF, which allows them to be computationally less expensive. To select features, ETCs use mean decrease impurity methods, which allow ranking of features in order of classification significance.

## 3. Results and Discussion

We performed the analysis by first calculating all sets of important features using each feature selection algorithm. Then, we analyzed the best-performing model for each feature selection algorithm, clinical test, and subject group. The features used were then compared by each best-performing model, as these features contain relevant information related to the falling problem. Furthermore, we compared these features across clinical tests. The ranking of features is different across feature selection algorithms as these use different criteria to rank features. Consequently, features found to be important across both feature selection mechanisms and both groups of subjects are discussed.

### 3.1. Top Features Selected by Both Feature Selection Algorithms for Each Subject Group

The sets of top 5, 10, 15, 20, 25, and 30 features (in descending order) selected by both feature selection algorithms for community-dwelling adults can be found in Table 3. As observed, for most clinical tests, the gait-related features are at the top of the table. This further indicates the importance of gait-related features to fall-risk screening, which is consistent with previous studies [70,71]. Moreover, features from the ML axis are predominant. This correlates with previous studies that present ML as indicative of fall risk in the elderly [72], irrespective of laboratory or clinical measures of postural stability.

The top features for stroke survivors are summarized in Table 4. Most gait-related features are at the top of the table, which highlights their importance for fall-risk assessment. For community-dwelling elderly, gait speed and step length are consistently within the top five important features. This is an indication that these two features might hold valuable information when studying falls across these two subject groups. Finding these features is also consistent with studies [73,74]. Moreover, most features for stroke survivors are related to the vertical axis, rather than the ML axis. We believe this is due to the need to maintain proprioceptive balance in stroke patients [75].

### 3.2. Best-Performing Models for Each Clinical Test, Feature Selection Mechanism, and Subject Group

The average AUC score for the best-performing models is highlighted in Table 5. In most cases, the best-performing models have a high AUC score, indicating an overall good classification performance. From the perspective of stroke survivors, the AUC of the TUG test is better for all sets of features. Similarly, from the perspective of community-dwelling elderly adults, almost all the best-performing models use the TUG test. Moreover, this table also shows that half of the best-performing models for stroke survivors use the set of top 30 features. While in contrast, half of the best models for community-dwelling elderly subjects use the set of top 15 features. This could be attributed to a larger number of samples in our community-dwelling elderly group. It can also be observed that in most cases, the models that use the multifactor clinical score as a label also use a smaller set of features than the models that use the SFBBS or TUG clinical scores. This can be explained by more robust classification criteria obtained after combining the SFBBS and TUG clinical scores. In addition, from the point of view of the feature selection mechanism, the AUC results of ETC are all higher than that of Relief-F, which is consistent with the findings of a similar study [60]. Moreover, as stated earlier, this study focuses on finding sets of features that can be used to study the underlying problems related to fall risk. Table 6 demonstrates that the trained models indeed are able to obtain good classification results. Thus, the features selected by these models are related to fall risk.

After finding the best model for each clinical test and each subject group, we also included other statistics from such models to show a more complete performance summary, as can be found in Table 6. When observing the results of using Relief-F as the feature selection mechanism, it can be observed that the AUC scores for TUG are the highest. Similarly, the precision results for community-dwelling elderly show that the TUG test obtained the best results since the features were directly extracted from the TUG acceleration signals. In contrast, when analyzing the F1 score, we can observe that the multifactor clinical test (SFBBS + TUG) had a better prediction accuracy for the stroke survivor subjects. Moreover, when analyzing the overall classification performance of using the features selected by ETC, we observed that TUG shows the best classification performance across both groups. This is expected as the features were extracted directly from the inertial acceleration data collected during the TUG test. Despite the higher AUC scores for most TUG tests, it is important to take into consideration that SFBBS and TUG tests measure different characteristics of a subject’s balance. TUG focuses on gait, while SFBBS focuses on static balance. Therefore, considering the multifactor test can provide a deeper and more robust understanding of a subject’s balance, as was suggested by previous studies [56,57].

### 3.3. Most Important Features for the Community-Dwelling Elderly and Stroke Survivors

From the community-dwelling elderly subjects, the set of repeated features that were found to be used by the best models of both feature extractor algorithms can be found in Table 7. As observed, only stand duration feature is present across all clinical tests (as it is highlighted by a “*”). This is consistent with previous studies, which found this feature to be significantly different between healthy and fall-risk subjects [53,76] and to be statistically significant between healthy elderly and those with transitional posture problems [54]. Meanwhile, gait speed was found to be important across two of the three clinical tests. This is consistent with previous studies that found this feature helpful for fall prediction [43,47].

From the stroke survivors, the set of repeated features that were used by the best models of both feature extractor algorithms is shown in Table 8. Two features were important across all three clinical tests, i.e., step length and stand duration (as highlighted by a “*”). Step length was also found in previous studies to predict falls [43] and was important to determine posture balance [77]. This can be related to gait speed, as subjects who have poor balance will try to maximize the time that they have for direct contact of their feet with the ground to avoid falling. Step length was important for fall-risk prediction among stroke survivors [74]. Moreover, duration of standing, step length, and stride time (important across two different clinical tests) were believed to be related to gait asymmetry and are related to fall risk [78] because they indicate the level of lower limb control the subject has while walking [79].

Finally, by analyzing the features found to be important across both groups (found in Table 9), it can be observed that four features were found to be important for the SFBBS clinical test, which is walk duration, gait speed, step length, and stand duration. Moreover, stand duration was found to be important for both the TUG test and the multifactor test. This can be attributed to a reduction and weakening of the hamstring muscles (located on the legs) of fall-risk individuals, which causes them to hastily perform the standing transitions [76]. Differences in the sit-to-stand transition can also be explained by a reduction in balance as the center of mass is raised further from the ground [37]. In summary, no matter which group of subjects is studied (community-dwelling or post-stroke), stand duration has important information that can help researchers and doctors to judge and further study fall risk among subjects.

Finding stand duration as a critical feature for posture assessment is backed by several studies which found it to be significantly different between healthy individuals and those at risk of falling [53,54,76,80]. Stand duration is calculated by measuring the total time in seconds it takes for the person to stand up from the sitting position. This time is calculated from the moment they lounge their upper body forwards to the moment they are standing upright, with legs fully stretched. In older individuals, a variation in stand duration can be explained by reduced muscle strength. This weakening of muscles results in a loss of balance during this transition which can lead to falls. In fact, clinical tests involving repeated sit-to-stand exercises have been found in previous studies to accurately identify individuals with reduced lower muscle strength [33,81]. Individuals with reduced muscle strength rely on their arms for support while standing, which increases the time they require to stand up [33].

According to multiple studies, post-stroke individuals are most susceptible to falling during sit-to-stand transitions [82,83,84]. This is generally caused by individuals shifting their weight towards their unaffected leg when standing up [57,82,85,86,87]. This shifting of weight causes individuals to require longer times to perform these transitions. Consequently, sit-to-stand tests have also been recommended as a tool to measure lower muscle strength in post-stroke individuals, as well as a screening tool for individuals at risk of falling [88,89].

Table 10 summarizes the values in seconds for stand duration obtained by each group of subjects. As observed, healthy individuals across all clinical tests were able to stand up in a shorter time when compared to individuals considered to be under fall risk, which suggests this feature can provide important information to identify subjects with postural problems. The values in seconds for the community elderly are consistent with a previous study that performed the TUG test on four different elderly subjects and measured the time it took individuals to stand up [80]. From this table, it can also be observed that stroke survivors that are not healthy (according to all clinical tests) have significant problems standing up compared to healthy stroke survivors as evident from the time they required to stand up.

## 4. Conclusions

This study analyzed the fall risk in individuals by automatically extracting features from the inertial sensor data collected from a TUG test and using machine learning to classify subjects as fallers and non-fallers. Our results show that the set of features extracted can provide good screening performance on either single or multifactor clinical tests. Using two feature selection algorithms, we found a set of important features, which were also found to be related to fall risk in previous studies.

We recognize there are some limitations with our study. Mainly, our subjects are mostly female, which makes it difficult to draw conclusions that can be representative of larger populations. However, due to the restrictions for recruitment which were necessary during our study as well as the difficulties involving recruiting individuals for medical studies, it was inevitable to recruit a balanced number of female and males. Nonetheless, this study provides a valuable contribution from the current perspective of technology-assisted scientific research.

Studies on fall-risk assessment are mostly limited by the type of subjects that participate in them. To address such limitation, this study is the first to compare the set of important features between two groups of subjects, which are the community-dwelling elderly and stroke survivors. By comparing these two groups, this study focused on finding a set of features that can be used to predict fall-risk, independent of the type of individuals being studied. Results showed that, across all clinical tests, only stand duration was found to be important to fall risk. This is consistent with a multitude of fall-risk assessment studies and is generally attributed to the weakening of muscles and the reduction in balance during such transition in subjects who are at risk of falling.

Finally, it is important to understand that the factors of fall risk are multiple, including muscle strength, cognitive function, environmental factors, etc. The purpose of this study is to use technology-assisted methods to focus on measuring acceleration data and pairing it with a clinical balance test task to find out the association between it and the risk of falling. Such an approach is valuable in studying the commonalities between specific eigenvalues and populations. Future studies could further explore other possible characteristics and factors to develop a more comprehensive and reliable fall-risk assessment model.

## Figures and Tables

**Table 1:** Demographic data for community-dwelling elderly subjects.

Subject Group	Description	Value	Number of Participants	Age (Mean ± STD)
Community-dwelling Elderly	Gender	Female	49	77 ± 6.60
		Male	16	73 ± 6.00
	Age	65–70	12	69 ± 1.72
		71–75	25	73 ± 1.53
		76–80	12	78 ± 1.48
		>80	16	85 ± 4.60
	Situation (SFBBS)	Healthy	61	72.64 ± 9.29
		Fall-Risk	13	78.5 ± 7.5
	Situation (TUG)	Healthy	61	71.91 ± 8.81
		Fall-Risk	13	81.61 ± 6.46
Stroke Survivors	Gender	Female	22	61 ± 11.78
		Male	13	61 ± 11.41
	Age	15–20	1	18 ± 0
		21–30	0	0
		31–40	4	34.5 ± 1.5
		41–50	2	46.5 ± 2.5
	Situation (SFBBS)	51–60	11	56 ± 3.23
		61–70	12	64 ± 1.32
	Situation (TUG)	71–80	4	73 ± 1.51
		>80	1	84 ± 0

**Table 2:** Features calculated from the inertial sensor data.

Feature Name (Feature Number)			
Full TUG Features			
MSE Mean (1–3)	MSE Standard Dev. (4–6)	MSE Complexity Idx. (7–9)	Permutation Ent. (10–12)
Sit to Stand (SiSt) Features			
Stand Duration (13)	Range (14–16)	Maximum Value (17–19)	Root Mean Square (20–22)
Maximum Jerk (23–25)	Minimum Value (26–28)	Mean Jerk (29–31)	Standard Dev. (32–34)
Walk Features			
Walk Duration (35)	Cadence (36)	Step Length (37)	Gait Speed (38)
Step Time (39)	Stride Time (40)	CV Step Time (41)	CV Stride Time (42)
Root Mean Square (43–45)			
Turn Features			
CV (46–48)	Median (49–51)	Range (52–54)	Root Mean Square (55–57)
Stand to Sit Features			
Sit Duration (58)	Range (59–61)	Root Mean Square (62–64)	Minimum (65–67)
Maximum (68–70)	Maximum Jerk (71–73)	Mean Jerk (74–76)	Standard Deviation (77–79)

**Table 3:** Top features selected by each feature selection algorithm for community-dwelling elderly.

Feature Name						
Top	Relief-F			ETC		
	y = SFBBS	y = TUG	y = SFBBS + TUG	y = SFBBS	y = TUG	y = SFBBS + TUG
1	Stand Duration	Stand Duration	Median Turn (ML)	Step Length	Gait Speed	Gait Speed
2	Gait Speed	RMS Turn (AP)	Range Turn (ML)	Gait Speed	Walk Duration	Walk Duration
3	Step Length	Min Stand (ML)	Stand Duration	Min Sit (ML)	Stand Duration	RMS Stand (ML)
4	Walk Duration	MSE Mean (AP)	Max Stand (AP)	RMS Walk (V)	Cadence	Std Stand (ML)
5	Std Stand (ML)	Jerk Stand (AP)	Range Sit (ML)	Walk Duration	RMS Walk (V)	Stand Duration
6	RMS Stand (ML)	RMS Stand (AP)	Min Sit (ML)	RMS Walk (ML)	Step Length	MSE Mean (AP)
7	MSE CI (ML)	Min Sit (V)	Gait Speed	MSE Mean (ML)	RMS Walk (ML)	Min Sit (ML)
8	MSE Mean (ML)	Max Stand (ML)	Median Turn (V)	MSE CI (ML)	Sit Duration	MSE CI (AP)
9	MaxJerk Stand V	MSE CI (AP)	Jerk Stand (AP)	RMS Stand (ML)	Step Time	RMS Walk (ML)
10	Stand Duration	Stand Duration	Median Turn (ML)	Step Length	Gait Speed	Gait Speed
11	CV Stride Time	PE (V)	Min Std (AP)	Std Stand (ML)	Stride Time	MSE Mean (ML)
12	CV Step Time	Median Turn (AP)	MaxJerk Stand AP	Stand Duration	RMS Stand (ML)	Step Length
13	Min Sit (ML)	Range Stand (V)	Std Sit (V)	MSE Std (AP)	Std Stand (ML)	MSE CI (ML)
14	MSE CI (V)	Std Stand (ML)	Jerk Stand (ML)	Min Stand (AP)	Std Sit (V)	Cadence
15	MSE Mean (V)	RMS Walk (V)	MaxJerk Sit (ML)	MSE CI (AP)	RMS Sit (V)	RMS Walk (V)
16	Median Turn (V)	RMS Walk (AP)	RMS Turn (V)	MSE CI (V)	MSE CI (ML)	PE (AP)
17	RMS Turn (V)	Std Stand (V)	MeanJerk Sit (ML)	MSE Mean (V)	MSE Mean (ML)	Step Time
18	RMS Walk (ML)	CV Turn (ML)	Max Stand (ML)	MSE Mean (AP)	Min Sit (ML)	Std Sit (V)
19	Max Stand (V)	MSE Mean (ML)	Range Turn (V)	Max Sit (V)	Range Sit (V)	RMS Sit (ML)
20	CV Turn (AP)	Range Sit (AP)	Max Stand (ML)	CV Turn (ML)	Std Sit (ML)	Std Sit (ML)
21	Median Turn (AP)	Gait Speed	RMS Walk (ML)	MaxJerk Stand AP	Median Turn (V)	RMS Sit (V)
22	PE (ML)	Min Stand (V)	CV Turn (AP)	Max Sit (V)	Min Stand (AP)	Sit Duration
23	Range Stand (ML)	MeanJerk Sit (AP)	RMS Sit (AP)	Range Stand (AP)	CV Stride Time	Stride Time
24	RMS Walk (V)	RMS Turn (V)	MSE CI (ML)	RMS Turn (AP)	Min Sit (V)	MaxJerk Stand AP
25	CV Turn (ML)	PE (AP)	PE (AP)	Range Stand (ML)	Range Stand AP	Range Stand ML
26	Max Stand (ML)	Step Time	Range Stand (ML)	MSE Std (ML)	CV Step Time	MSE Mean (V)
27	PE (V)	Min Std (AP)	Range Sit (V)	Range Sit (AP)	Range Stand ML	MSE CI (V)
28	Min Stand (ML)	Median Turn (ML)	Jerk Stand (V)	Jerk Stand (ML)	MSE Std (V)	Range Sit (AP)
29	Max Stand (ML)	RMS Sit (AP)	Range Stand (AP)	Jerk Stand (V)	Min Stand (ML)	Min Std (AP)
30	RMS Turn (AP)	Range Turn (ML)	Max Sit (V)	Median Turn (V)	Range Sit (ML)	MSE Std (AP)

**Table 4:** Top features selected by each feature selection algorithm for stroke survivors.

Feature Name						
Top	Relief-F			ETC		
	y = SFBBS	y = TUG	y = SFBBS + TUG	y = SFBBS	y = TUG	y = SFBBS + TUG
1	Walk Duration	MSE Mean (V)	Std Stand (ML)	Step Length	Gait Speed	Step Length
2	Gait Speed	RMS Sit (V)	Std Sit (AP)	Gait Speed	Step Length	Gait Speed
3	Step Length	MSE Std (ML)	Median Turn (AP)	Walk Duration	Walk Duration	Walk Duration
4	CV Step Time	Min Std (AP)	Min Sit (V)	CV Step Time	Cadence	MSE Std (ML)
5	CV Stride Time	RMS Walk (ML)	Range Stand (ML)	RMS Walk (ML)	MSE Std (ML)	RMS Walk ML
6	RMS Walk (ML)	CV Step Time	MSE Std (V)	CV Stride Time	MaxJerk Stand AP	Sit Duration
7	Sit Duration	PE_V	RMS Turn (AP)	MSE Std (ML)	Step Time	CV Step Time
8	Cadence	Stand Duration	Max Sit (AP)	Sit Duration	Min Stand (AP)	Cadence
9	Std Sit (AP)	Std Sit (V)	RMS Turn (V)	RMS Turn (V)	RMS Stand (ML)	Std Sit (AP)
10	Median Turn (AP)	Min Std (AP)	MSE Mean (V)	CV Turn (AP)	Jerk Sit (ML)	Stand Duration
11	Step Time	Range Sit (ML)	Max Sit (V)	Std Sit (AP)	Stride Time	CV Stride Time
12	Stride Time	MSE CI (V)	RMS Stand (V)	MaxJerk Sit (AP)	Stand Duration	Stride Time
13	Stand Duration	Min Stand (AP)	CV Turn (V)	RMS Walk (AP)	RMS Stand (AP)	Step Time
14	MaxJerk Sit (AP)	MaxJerk Sit (ML)	Gait Speed	Cadence	Range Stand (AP)	MSE Std (V)
15	RMS Turn (V)	Range Sit (V)	RMS Stand (ML)	Std Sit (V)	Std Stand (AP)	RMS Turn (V)
16	MSE Std (ML)	Median Turn (ML)	Cadence	Step Time	MSE Std (V)	Jerk Sit (AP)
17	Median Turn (V)	Sit Duration	Stand Duration	PE (AP)	Std Stand (ML)	RMS Walk (AP)
18	Range Sit (ML)	Median Turn (V)	Min Sit (ML)	Jerk Sit (AP)	MaxJerk Stand V	Jerk Stand (V)
19	RMS Walk (AP)	Step Length	MaxJerk Sit (ML)	Stride Time	Jerk stand ML	Std Sit (V)
20	Min Sit (ML)	Stride Time	Max Stand (ML)	Stand Duration	Std Sit (AP)	MSE Mean (V)
21	Std Sit (V)	MaxJerk Stand AP	RMS Sit (V)	Median Turn (AP)	MaxJerk Sit (ML)	PE (AP)
22	MaxJerk Sit (ML)	RMS Walk (AP)	Jerk Stand V	Jerk Sit (V)	RMS Walk (ML)	MSE CI (V)
23	Range Sit (AP)	Range Turn (ML)	Min Stand (ML)	Max Sit (V)	Max Stand (AP)	CV Turn (AP)
24	CV Turn (AP)	RMS Turn (V)	Step Time	Median Turn (V)	Range Turn (V)	Median Turn AP
25	Jerk Stand ML	RMS Stand (ML)	CV Turn (AP)	MSE CI (V)	RMS Sit (AP)	Jerk Sit (ML)
26	RMS Sit (V)	Median Turn (AP)	Jerk Sit (V)	MSE Mean (V)	Max Stand (ML)	Std Sit (ML)
27	Median Turn (ML)	Std Stand (ML)	Max Stand (V)	Jerk Stand (V)	Max Sit (V)	Max Sit (V)
28	MSE CI (V)	Min Sit (V)	MaxJerk Sit (AP)	MSE Std (V)	CV Turn (AP)	RMS Walk (V)
29	MSE Mean (V)	Std Sit (AP)	Range Turn (ML)	Jerk Stand ML	Min Std (AP)	Median Turn V
30	RMS Sit (AP)	MSE Std (V)	Std Sit (ML)	MaxJerk Stand AP	MSE CI (AP)	Jerk Sit (V)

**Table 5:** Summary of AUC average scores for all models.

	Subjects	y	Top 5	Top 10	Top 15	Top 20	Top 25	Top 30
Relief-F	Stroke Survivors	SFBBS	0.789	0.781	0.781	0.790	0.785	0.798
		TUG	0.808	0.855	0.864	0.967	0.965	0.956
		SFBBS + TUG	0.609	0.715	0.805	0.810	0.835	0.838
	Community Dwelling Elderly	SFBBS	0.834	0.820	0.843	0.824	0.802	0.810
		TUG	0.869	0.870	0.919	0.983	0.986	0.987
		SFBBS + TUG	0.829	0.945	0.949	0.941	0.941	0.940
ETC	Stroke Survivors	SFBBS	0.815	0.834	0.829	0.838	0.835	0.849
		TUG	0.990	0.993	0.994	0.994	0.995	0.995
		SFBBS + TUG	0.922	0.927	0.932	0.932	0.918	0.931
	Community Dwelling Elderly	SFBBS	0.850	0.867	0.870	0.869	0.870	0.866
		TUG	0.991	0.995	0.995	0.993	0.994	0.994
		SFBBS + TUG	0.962	0.975	0.970	0.965	0.954	0.962

**Table 6:** Performance statistics for the best models.

	Subjects	y	AUC	Precision	Recall	F1-Score
Relief-F	Stroke Survivors	SFBBS	0.798	0.742	0.716	0.729
		TUG	0.967	0.787	0.815	0.801
		SFBBS + TUG	0.838	0.814	0.795	0.804
	Community Dwelling Elderly	SFBBS	0.843	0.761	0.810	0.785
		TUG	0.987	0.949	0.944	0.946
		SFBBS + TUG	0.949	0.841	0.912	0.875
ETC	Stroke Survivors	SFBBS	0.849	0.794	0.768	0.781
		TUG	0.995	0.976	0.969	0.972
		SFBBS + TUG	0.932	0.854	0.831	0.842
	Community Dwelling Elderly	SFBBS	0.870	0.770	0.811	0.790
		TUG	0.995	0.989	0.987	0.988
		SFBBS + TUG	0.975	0.890	0.923	0.906

**Table 7:** Features selected by the best models for the community-dwelling elderly. Features found to be repeated across all clinical tests are marked with a “*”.

SFBBS	TUG	SFBBS + TUG
Walk Duration	Stand Duration *	Gait Speed
Gait Speed		Stand Duration *
Step Length		
Stand Duration *		
MSE-V CI		
MSE-V Mean		

**Table 8:** Features selected by the best models for the stroke-survivors. Features found to be repeated across all clinical tests are marked with a “*”.

SFBBS	TUG	SFBBS + TUG
Walk duration	Stand duration *	Walk duration
Gait speed	Step Length *	Sit duration
Step length *		Stride time
CV step time		Mean sit (V)
RMS walk (ML)		Stand duration *
CV stride time		CV stride time
Sit duration		Step Length *
Std sit (AP)		
RMS turn (Y)		
Jerk sit (AP)		
Cadence		
Median turn (AP)		
Stand duration *		
Step time		
Stride time		

**Table 9:** Most important features across both groups of subjects.

SFBBS	TUG	SFBBS + TUG
Walk Duration	Stand Duration	Stand Duration
Gait Speed		
Step Length		
Stand Duration		

**Table 10:** Table summarizing the stand duration (in seconds) for all subjects in our study, organized according to their situation and their clinical test score.

Subjects	Situation	BBS Mean (SD)	TUG Mean (SD)	BBS + TUG Mean (SD)
Stroke Survivors	Fall Risk	4.14 (1.76)	3.59 (1.74)	4.58 (2.08)
	Healthy	1.81 (0.85)	1.27 (0.25)	1.27 (0.82)
Community Dwelling Elderly	Fall Risk	1.37 (0.46)	1.70 (0.37)	1.66 (0.32)
	Healthy	1.07 (0.39)	1.00 (0.30)	0.99 (0.39)

## Data Availability

The data presented in this study are available on request from the corresponding author. The data are not publicly available due to it belonging to individuals who participated in the study.
